# Use of point-of-care glucometers during an oral glucose tolerance test in children for prediabetes and diabetes diagnosis: a comparison study

**DOI:** 10.1515/almed-2023-0089

**Published:** 2023-11-29

**Authors:** Blanca Fabre-Estremera, Estéfani Martínez-Chávez, Marta Manzano Ocaña, Atilano Carcavilla Urquí, María de los Ángeles Morales Sánchez, Inmaculada Pinilla Tejado, Isabel González-Casado, Itsaso Losantos García, Pilar Fernández-Calle, Antonio Buño Soto, Paloma Oliver

**Affiliations:** Department of Laboratory Medicine, La Paz University Hospital, Madrid, Spain; Department of Pediatrics Endocrinology, La Paz University Hospital, Madrid, Spain; Department of Biostatistics, La Paz University Hospital, Madrid, Spain

**Keywords:** diabetes mellitus, glucose tolerance test, point-of-care testing

## Abstract

**Objectives:**

Despite clinical guidelines do not recommend the use of point-of-care testing (POCT) glucometers for diagnostic purposes yet, the analytical performance is continuously improving. Thus, we evaluate the technical accuracy and clinical concordance of POCT glucometers during an oral glucose tolerance test (OGTT) in children for prediabetes and diabetes diagnosis in a comparison study.

**Methods:**

Pediatric patients with an OGTT indication who attended the Diabetes Unit between December 2020 and September 2021 were recruited for this prospective observational study. During the functional test, glycaemia was immediately measured in venous blood using two glucometers (unconnected and connected) and sent to the central laboratory.

**Results:**

The study included 98 patients. There was a high correlation between the glucometers and the central laboratory (Pearson correlation coefficient=0.912 and 0.950, for unconnected and connected glucometer, respectively). The median OGTT turnaround time (TAT) was significantly decreased (connected glucometer: 2.02 h [interquartile range, 2.00–2.07], central laboratory: 11.63 h [6.09–25.80]), with similar overall cost. The diagnostic concordance between connected glucometer and the central laboratory was 71.1 % (95 % confidence interval (CI) 61.5–79.2). The clinical decision would have been the same in the 92.8 % of the cases, but treatment would have not been indicated in 4 patients (4.1 %).

**Conclusions:**

POCT glucometers have demonstrated a high correlation and an acceptable diagnostic concordance with the central laboratory during an OGTT, as well the connected device offers a significant decrease in TAT, without increasing costs. However, as severe clinical impact could happen, POCT glucometers may not be used for diagnosis yet.

## Introduction

Accurate measurement of glycaemia is essential for diagnosing diabetes and prediabetes [1]. The American Diabetes Association (ADA) defines the criteria for diabetes and prediabetes based on glucose concentration and the presence or absence of symptoms [1, 2]. Glycaemia can be measured in a central laboratory or through point-of-care testing (POCT), a set of tests designed for use at or near the patient. Despite the improvements in analytical performance of POCT glucometers, their use for diagnostic purposes has not yet been generally accepted [1, 3]. POCT platforms offer several advantages compared to central laboratory testing. In addition to providing results rapidly, POCT devices have small specimen volume requirements compared to central laboratory tests making POCT particularly attractive for pediatric healthcare settings.

Some studies have evaluated the diagnostic agreement of glucometers with the central laboratory during oral glucose tolerance test (OGTT) [4], [5], [6]. To the best of our knowledge, the published studies have not included pediatric populations and they used capillary blood instead of venous plasma [4], [5], [6], as recommended by clinical diabetes guidelines [1, 3]. Limited evidence and the discrepancies in some results warrant further investigation [4], [5], [6].

As we have been performed in other contexts previously [7], [8], [9], we aimed to evaluate the technical accuracy and clinical concordance of POCT glucometers during OGTT on pediatric patients. It was evaluated considering the following objectives: glycaemia correlation, the progression of glucose measurements during OGTT, the turnaround time (TAT), diagnostic concordance and costs.

## Materials and methods

### Study design and participants

The Laboratory Medicine Department of La Paz University Hospital is accredited by ISO 15,189 and has led a multiparameter and multisite POCT network over the last 23 years and it is accredited by ISO 22,870.

We conducted a prospective observational study that invited pediatric patients with an OGTT indication who visited the Diabetes Unit of the Pediatric Endocrinology Department to participate from December 2020 to September 2021. It was approved by the Clinical Research Ethics Committee of our hospital (#4,358), and written informed consent was obtained from all participants.

The sample size was calculated using the formula for equivalence studies. A maximum error of 4 mg/dL was established between the glucose values (SI units, mmol/L; conversion factor, 0.0555), from the central laboratory and glucometers, with a common variance for the glucose values of 20. With a significance of 0.05 and a statistical power of 0.90, the sample size estimated was 81 patients.

**Figure 1:**
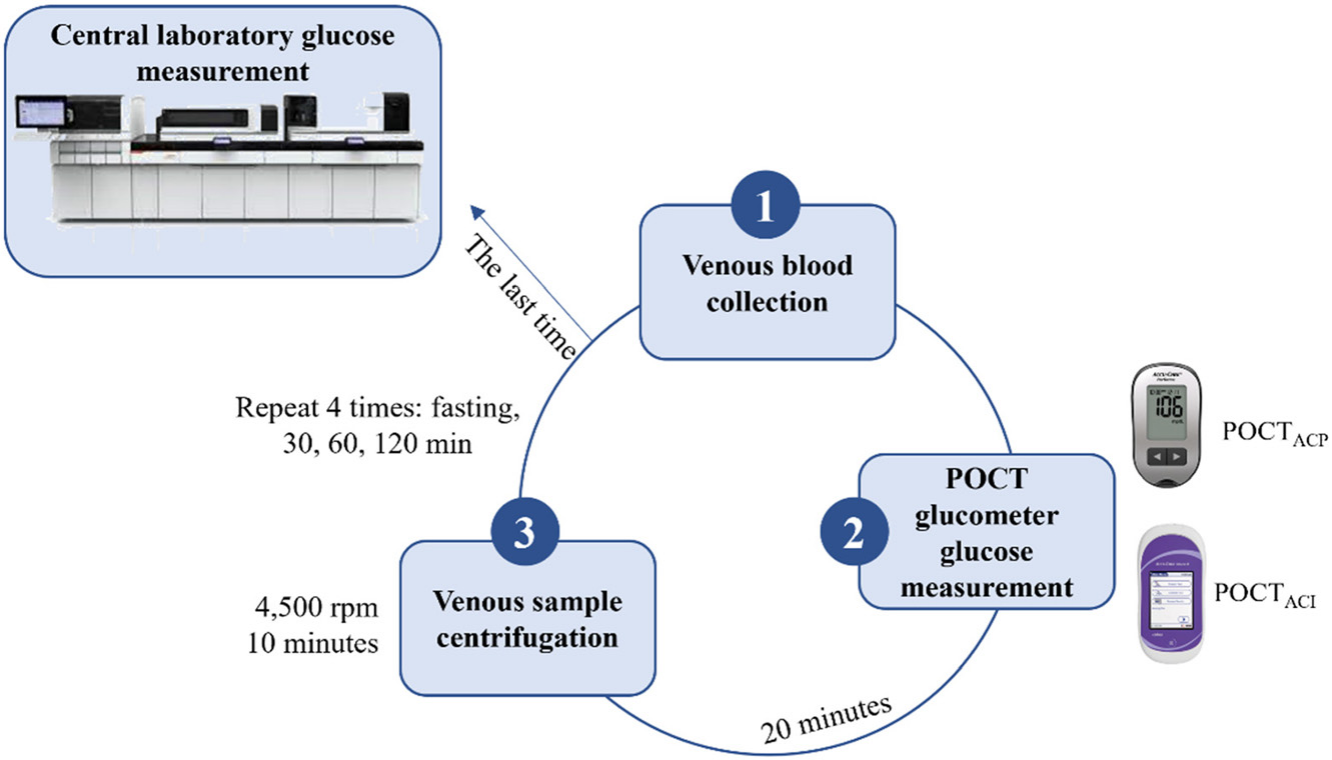
Analytical measurement for the glucose readings during the study period. POCT_ACI_, connected glucometer; POCT_ACP_, unconnected glucometer.

At each venous blood collection during OGTT, whole blood glucose was measured immediately with two glucometers. Venous samples were collected in serum gel tubes (BD Vacutainer, Mexico City, Mexico). After 20 min, the samples were centrifuged at 4,500 rpm for 10 min at the Diabetes Unit and kept at room temperature. This cycle was repeated with each extraction (fasting, at 30, 60 and 120 min). At the end of the OGTT, the samples were sent to the central laboratory for their analysis (Figure 1).

To examine the technical accuracy and clinical concordance of glucometers during OGTT, two glucometers, both cleared for professional use by the FDA [10] and used for clinical decision making in our hospital, were evaluated, considering the central laboratory as reference:

– Accu-Chek^®^ Performa (Roche Diagnostics, Basel, Switzerland): POCT_ACP_, unconnected, enzymatic method (quinoprotein glucose dehydrogenase), venous sample [whole blood, automatic conversion factor to plasma (11)]. The inter-assay coefficients of variation (CVs) were 2.25 and 1.45 % of internal quality control (IQC), for a mean concentration of 46 and 307 mg/dL, respectively.
– Accu-Chek^®^ Inform-II (Roche Diagnostics, Basel, Switzerland): POCT_ACI_, connected, enzymatic method (quinoprotein glucose dehydrogenase), venous sample (whole blood, converted to plasma [11]). The CVs were 1.75 and 1.67 %, for 45 and 303 mg/dL of IQC, respectively.
– Atellica^®^ Solution-CH (Siemens Healthineers, Erlangen, Germany): central laboratory, enzymatic method (glucose hexokinase), venous sample (serum). The CVs were 1.96 and 2.17 %, for 59 and 343 mg/dL of IQC, respectively. It is used for diagnosis during OGTT.

The times and results from the POCT_ACP_ were collected in a manual register, according to daily clinical practice. Those from the POCT_ACI_ and the central laboratory were obtained from the laboratory information system (LabTrak; Intersystems, Cambridge, MA, USA).

### Correlation of glucose measurements during OGTT

A previous comparative study was carried out between the three analyzers for glucose measurements based on the Clinical and Laboratory Standards Institute EP09 [12]. 3 samples were processed in duplicate (glucose concentrations from 100 to 222 mg/dL) on each analyzer for 3 consecutive days, demonstrating the interchangeability of patient’s results at the clinical decision levels.

The correlation was evaluated at fasting, and at 30, 60 and 120 min. Methods comparison between POCT_ACI_ and the central laboratory was carried out.

### Turnaround time

We considered the time elapsed during OGTT, from the first blood sample collection to the time when all results were available for clinical staff, as the TAT.

### Diagnostic concordance

The diagnostic concordance between the glucometers and the central laboratory (as a reference) was assessed according to the American Diabetes Association (ADA) criteria (Supplementary Material Table S1) [1].

Analytical performance specifications were based on biological variation [13]. Total allowable error (TE) was calculated by following equation [14]:

$$\text{TE}<1.65\times \left(0.5\times {\text{CV}}_{I}\right)+0.25\times {\left({\text{CV}}_{I}^{2}+{\text{CV}}_{G}^{2}\right)}^{1/2}$$

CV_I_: 4.9 %, CV_G_: 8.1 % [13].

The factor 0.5 refers to a desirable specification.

CV_I_, intraindividual coefficient of variation; CV_G_, interindividual coefficient of variation.

Therefore, we established the TE of 6.4 %, based on desirable specification as we used in our laboratory. In case of diagnostic discrepancies between POCT_ACI_ and the central laboratory, we calculated the percentage differences, and cases that exceeded our specification were analyzed.

### Costs

Overall costs during the study period were evaluated considering the material resources related to each analyzer, and the staff cost was provided by the Department of Human Resources. An estimate of the staff’s work time for each patient during OGTT was considered. The remaining costs were provided by the Department of Supplies and the Central Services Administrative Unit.

### Connectivity

As POCT_ACP_ and POCT_ACI_ is an unconnected and connected glucometer, respectively, we considered the transcription errors during the study period to evaluate the postanalytical phase. Additionally, we administered a satisfaction survey to the clinical staff to consider satisfaction of connectivity as an explorative objective (Supplementary Material File S1).

### Statistical analysis

Qualitative data was described with absolute frequencies and percentages and the quantitative data as mean and standard deviation (SD) or as median and interquartile range (IQR) depending on the distribution of these data.

The normality of continuous variables was studied using the Kolmogorov-Smirnov test.

The association between glucose concentrations was studied using Pearson’s correlation. For the comparison of glucose concentrations and TAT, we employed Student’s t-test and Wilcoxon test, respectively. The diagnostic concordance was examined using the percentage of observed agreement with their 95 % confidence intervals (95 % CI).

All statistical tests were considered bilateral, and those with an error probability less than 5 % (p<0.05) were considered statistically significant. The data were analyzed with the statistical program SAS 9.3 (SAS Institute, Cary, NC, USA).

## Results

112 pediatric patients were indicated for OGTT, 7 of whom did not sign the informed consent. We excluded 7 participants who did not finish the OGTT, resulting in a final study sample of 98 participants (Supplementary Material Figure S1). Of these, 53.1 % were girls, and the median (IQR) age was 12 years (10–14).

### Correlation of glucose measurements during OGTT

The glucose concentrations obtained by the three analyzers, and their Pearson’s correlation, are summarized in Tables 1 and 2. Statistically significant differences were observed between the mean values in fasting, glucose concentrations were higher with POCT glucometers.

**Table 1:** Glucose concentration (mg/dL) during oral glucose tolerance testing.

	POCT_ACP_	POCT_ACI_	Central laboratory
Fasting	96 (8.3)^a^	96 (8.5)^a^	92 (9.0)
At 30 min	150 (26.5)	150 (28.6)	156 (28.6)
At 60 min	151 (37.3)	151 (37.1)	161 (45.9)
At 120 min	133 (33.0)	133 (32.3)	139 (36.9)

Mean (SD); ^a^p<0.05. POCT_ACI_, connected glucometer; POCT_ACP_, unconnected glucometer; SD, standard deviation.

**Table 2:** Correlation of glucose measurements between POCT glucometers and central laboratory.

		POCT_ACP_	POCT_ACI_
		Pearson correlation coefficients	
Central laboratory	Fasting	0.787^a^	0.786^a^
	At 30 min	0.633^a^	0.937^a^
	At 60 min	0.878^a^	0.876^a^
	At 120 min	0.984^a^	0.980^a^
	Overall	0.912^a^	0.950^a^

a p<0.001. POCT_ACI_, connected glucometer; POCT_ACP_, unconnected glucometer.

Supplementary Material File S2 shows the methods comparison between POCT_ACI_ and the central laboratory.

### Turnaround time

**Figure 2:**
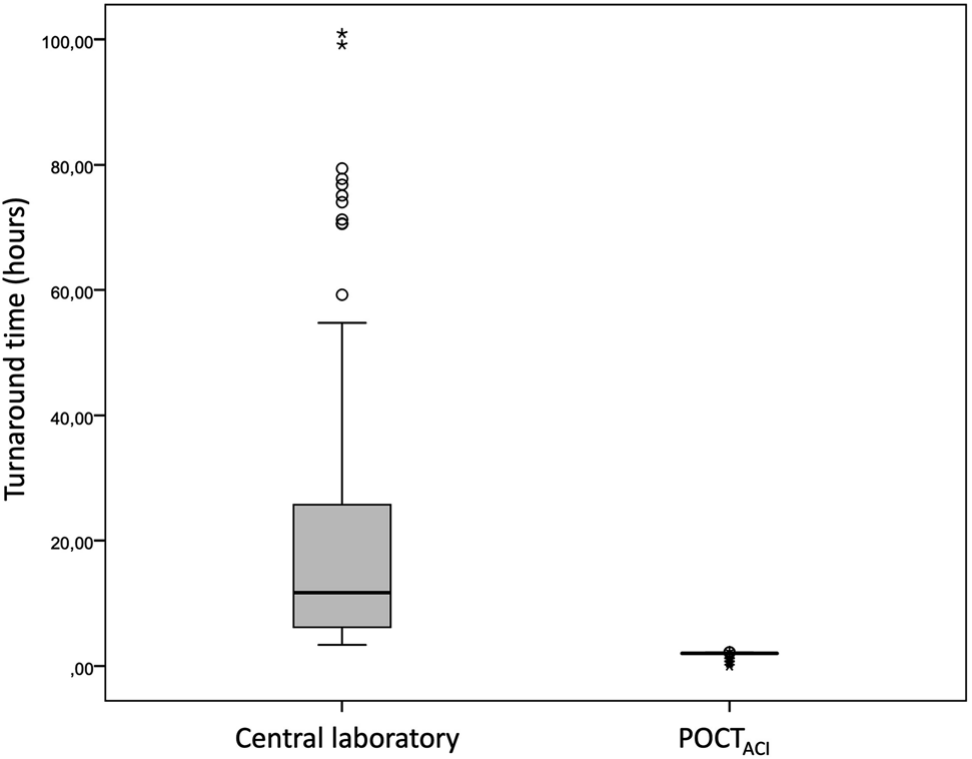
Turnaround time for oral glucose tolerance testing using POCT glucometers vs. the central laboratory. POCT_ACI_, connected glucometer.

There were statistically significant differences between the median (IQR) of TATs for OGTT of the connected glucometer and the central laboratory (2.02 h (2.00–2.07) and 11.63 h (6.09–25.80), respectively, p<0.001) (Figure 2). The unconnected glucometer involves a higher TAT compared to the connected glucometer, due to the transcription during the workday of the results to the patient’s electronic medical record.

### Diagnostic concordance

The diagnostic concordance between the glucometers and the central laboratory was 74 % (95 % CI 69.4–81.7) for POCT_ACP_ and 71.1 % (95 % CI 61.5–79.2) for POCT_ACI_ (Table 3).

**Table 3:** Diagnostic concordance between the POCT glucometers and the central laboratory.

		Central laboratory				
		Normal	IFG	IGT	IFG+IGT	Diabetes
**POCT**_**ACP**_ **, n (%)**	**Normal**	**44 (80 %)**		6 (28.6 %)		
	**IFG**	10 (18.2 %)	**5 (100 %)**	2 (9.5 %)	1 (12.5 %)	
	**IGT**	1 (1.8 %)		**11 (52.4 %)**	1 (12.5 %)	2 (28.6 %)
	**IFG+IGT**			2 (9.5 %)	**6 (75 %)**	
	**Diabetes**					**5 (71.4 %)**
**POCT**_**ACI**_ **, n (%)**	**Normal**	**44 (80 %)**	1 (20 %)	7 (33.3 %)		
	**IFG**	10 (18.2 %)	**4 (80 %)**	1 (4.8 %)	1 (12.5 %)	
	**IGT**	1 (1.8 %)		**12 (57.1 %)**	1 (12.5 %)	4 (50.0 %)
	**IFG+IGT**			1 (4.8 %)	**6 (75 %)**	1 (12.5 %)
	**Diabetes**					**3 (37.5 %)**

IFG, impaired fasting glucose; IGT, impaired glucose tolerance; POCT_ACI_, connected glucometer; POCT_ACP_, unconnected glucometer. The bold values refer to the diagnostic agreement between the glucometers and the central laboratory in each clinical entity during oral glucose tolerance test.

In the discordant cases, we calculated the percentage differences in glucose concentrations between the glucometers and the central laboratory. POCT_ACI_ had more cases (11 cases) with percentage differences smaller than our specification (TE=6.4 %) than POCT_ACP_ (7 cases). Initially, although POCT_ACI_ appeared to have lower concordance than POCT_ACP_, when we recalculated the diagnostic agreement including cases that did not exceed our specification, POCT_ACI_ ultimately had slightly higher diagnostic concordance (82.5 %) than POCT_ACP_ (81.3 %).

These diagnostic discrepancies between POCT_ACI_ and the central laboratory were evaluated individually and classified as having no clinical impact, mild clinical impact (indication for glycemic control or repetition of the OGTT) or severe clinical impact (indication for treatment) (Table 4).

**Table 4:** Diagnostic discrepancies between POCT_ACI_ and the central laboratory.

POCT_ACI_	Central laboratory	Comment	Clinical impact
IFG	Normal		None
IFG	Normal		
IFG	Normal		
IFG	Normal		
IFG	Normal		
IFG	Normal		
IFG	Normal	CF – AIG	
Normal	IGT	CF – AIG	
IFG	IFG+IGT	CF	
IGT	Diabetes	CF	
Normal	IGT		Mild
IGT	Diabetes	CF – no diagnosis of diabetes	
IGT	Diabetes	No diagnosis of diabetes	
Normal	IGT		Severe
Normal	IGT		
IFG+IGT	Diabetes		
IGT	Diabetes	Repetition OGTT – no diagnosis of diabetes	

AIG, indeterminate glucose alteration; CF, patient with cystic fibrosis; IFG, impaired fasting glucose; IGT, impaired glucose tolerance; POCT_ACI_, connected glucometer.

### Costs

Supplementary Material Table S2 summarizes the costs associated with the processes during the study period for each analyzer. The overall cost per process with the POCT devices was lower than those with the central laboratory devices.

### Connectivity

During the study period, we reported a 1.8 % rate of transcription errors. While the transcription errors we identified during the study period would not have led to a change in diagnosis, it is important to note that the fact that they did not have consequences in our study period does not diminish the potential risk of an error can affect the clinical management of patients.

Questionnaires were given to 6 clinicians and 4 nurses, all of whom recommended incorporating POCT_ACI_ into similar clinical scenarios. The mean score was 4.7 (1=worst, 5=best).

## Discussion

### Correlation of glucose measurements during OGTT

We observed a high correlation between the POCT glucometers and the central laboratory and no statistically significant differences were found, except at fasting when the glucose concentrations were higher with the POCT glucometers. Reviewing our clinical procedure, we identified that fasting samples were not centrifuged immediately after blood clotting at the Diabetes Unit. Those samples were centrifuged together with the samples at 30 min. The differences found could be explained by *in vitro* glycolysis, which could lead to results from the glucometers that are more consistent at fasting. The glycolysis rate has been estimated at 5–7% (3–4 mg/dL in 30 min), similar to our results [15]. After the end of this study, we changed the clinical procedure so that currently, fasting samples are also centrifuged immediately after blood clotting. A later evaluation of these results during the following six months after the study revealed no statistically significant differences of fasting results.

### Turnaround time

The significant decrease in TAT when only POCT devices are used has already been demonstrated in numerous clinical settings [7, 9, 16]. In our investigation, not only each glucose measurement improved considerably its TAT but also OGTT by POCT_ACI_, an important finding because the results are immediately available, allowing clinicians to make decisions after the OGTT and avoiding the need for additional patient visits. Additionally, if we assume that transcription of glucose results takes 4 min per OGTT, POCT_ACI_ would have saved almost 7 h for the nurses during the study period.

### Diagnostic concordance

OGTT using a venous sample is the gold standard for diagnosing diabetes and detecting prediabetes via impaired fasting glucose (IFG) and impaired glucose tolerance (IGT), which are the intermediate stages between normal glucose homeostasis and diabetes. These stages are not clinical entities but indicate a relatively high risk of developing diabetes and cardiovascular disease, especially in the context of obesity [3].

All measurements have analytical and biological variability. It is therefore possible that an abnormal result, when repeated, will produce a value below the diagnostic cut-off and vice versa. Even with an acceptable analytical performance, low analytical imprecision around the diagnostic cut-off could lead to a different classification. Clinicians are advised to discuss the signs and symptoms with the patient and consider repeating the test [1].

Our results showed an acceptable global diagnostic concordance, consistent with previous studies which used capillary and venous blood, respectively [6, 17]. Though, for the diabetes category, we observed higher discrepancies.

Analyzing the clinical impact of the diagnostic discrepancies between POCT_ACI_ and the central laboratory, we found that in the group with no clinical impact, several patients had cystic fibrosis. Currently, OGTT is the most sensitive screening test for detecting diabetes secondary to cystic fibrosis for which, in addition to other clinical entities, indeterminate glucose disorder is added (fasting plasma glucose (FPG)<126 mg/dL, 2 h OGTT<140 mg/dL and OGTT≥200 mg/dL at 30, 60 or 90 min) [18]. In most patients, all analyzers diagnosed an indeterminate glucose disorder. In addition, it is important to emphasize that most of the discrepancies in this group corresponded to patients diagnosed with IFG by glucometers and with normal OGTT results according to the central laboratory. These differences could be explained by *in vitro* glycolysis in the fasting samples, as stated previously. Such decreases in glucose concentration could lead to missing IFG diagnoses in the proportion of patients with glucose concentrations near the cut-off points. If the immediate centrifugation of the fasting samples had been performed, these discrepancies might not have been observed.

In all cases with mild clinical impact, the diagnostic discrepancy is produced by the proximity of the results to the cut-off points. In fact, although the results from the central laboratory met the criteria for diabetes in two of the three cases, only glycemic control and repetition of the OGTT were indicated.

Among the patients with severe clinical impact, one patient whose results from the central laboratory met the criteria for diabetes was indicated treatment, but the diagnosis of the disease was not achieved, leading to the repetition of the OGTT.

### Costs

It is important to note that the cost per measurement with a POCT device is normally higher. However, if we consider all the issues involved, the overall cost per process could be similar or even lower than those of the central laboratory [7, 9, 19]. Our results are in line with those findings, with a higher glucose measurement cost when using POCT_ACI_ than with the central laboratory, while the overall cost by process per 98 patients was similar. Additionally, other studies in other clinical contexts have found that POCT could lead not only to clinical effectiveness but also cost-effectiveness [19, 20].

### Connectivity

The ISO 15,189 standard specific for POCT recommends implementing connectivity whenever possible [21]. To our knowledge, this is the first study to consider a connected glucometer from a POCT network accredited by ISO 22,870 compared with a central laboratory. Previous studies have emphasized that connectivity is the safest method for data transfer, avoiding postanalytical errors related to manual entry [22, 23]. Mays et al. reported a higher rate of discrepancies of 3.7 % for manual data transfer, which were clinically significant in 5 of every 1,000 results [22]. We observed a rate of 1.8 % of transcription errors in our study with the non-connected glucometer. This finding enhances the need of connectivity. An education intervention for clinical staff coupled with an online quality control program allowed to reduce post-analytical errors [24].

We have evidence the benefits of connectivity considering the staff satisfaction, an important issue in POCT when introducing this activity in clinical practice. The evidence suggests that satisfaction is linked to improved care and greater adherence to management recommendations [25, 26]. Our study confirmed the fact that clinical staff saw substantial advantages in using connected glucometers.

### Strengths and limitations

Our study has several strengths. Despite the glucometers have not yet been generally accepted for diagnosis and we still have to wait for the guidelines recommendations, this is the first study that compares the use of glucometers from a POCT network accredited by ISO 22,870 and central laboratory accredited by ISO 15,189 in a pediatric population. Its major strength is the prospective design, which included both POCT_ACP_ and POCT_ACI_. Another strength is that our study considered not only the diagnostic concordance but also the clinical impact. However, we need to acknowledge certain limitation, such as limited sample size and the influence of *in vitro* glycolysis in our results. Lastly, our results might not be generalizable to adults or pregnant women.

## Conclusions

POCT glucometers have demonstrated a high correlation with the central laboratory during OGTT in pediatric patients, as well as offers a significant decrease in TAT, without increasing costs. The diagnostic concordance with the central laboratory has not resulted as high as desired, although the clinical decision would have been the same in the 92.8 % of the cases. Consequently, and as the clinical guidelines recommend, POCT glucometers may not be used for diagnosis yet, remaining them as a support tool for the central laboratory during OGTT.

## Supplementary Material

Supplementary Material
