# Supplementary material for: Use of point-of-care glucometers during an oral glucose tolerance test in children for prediabetes and diabetes diagnosis: a comparison study
Source: Adv Lab Med. 2023 Nov 29;5(2):189–96. doi: 10.1515/almed-2023-0089 (PMC11206188; doi:10.1515/almed-2023-0089)
Supplement: Supplementary file 1 — Supplementary Material [file j_almed-2023-0089_suppl_001.docx]

**Supplemental Data Table S1.** Clinical entities during oral glucose tolerance test (1)

|  | **Glucose concentration** |
| --- | --- |
| **IFG** | FPG, 100–125 mg/dL |
| **IGT** | 2-h OGTT, 140–199 mg/dL |
| **IFG+IGT** | FPG, 100–125 mg/dL and  2-h OGTT, 140–199 mg/dL |
| **Diabetes** | FPG ≥126 mg/dL or  2-h OGTT ≥200 mg/dL |

Abbreviations: FPG, fasting plasma glucose; IFG, impaired fasting glucose; IGT, impaired glucose tolerance; OGTT, oral glucose tolerance test

**Supplemental Data Table S2.** Overall cost per process when using each analyzer

|  |  | Cost per issue (€) | Number of issues per patient | Total costs (€) | Total costs n=98 |
| --- | --- | --- | --- | --- | --- |
| **Common costs** | Lab doctor’s working hours | 34.84 | 0.01 | 34.14 |  |
|  | Endocrinology doctor’s working hours | 34.84 | 0.01 | 34.14 |  |
|  | Nurse’s working hours | 20.34 | 2.00 | 3986.64 |  |
|  | Glucose bolus | 0.76 | 1 | 74.48 |  |
| **POCT_ACP_** | Specimen collection | 2.47 | 1 | 235.2 | **€4400.86** |
|  | Material for glucose measurement | 0.37 | 1 | 36.26 |  |
| **POCT_ACI_** | Specimen collection | 2.47 | 1 | 235.2 | **€4431.24** |
|  | Material for glucose measurements | 0.68 | 1 | 66.64 |  |
| **Central laboratory** | Specimen collection | 3.06 | 1 | 299.88 | **€4469.31** |
|  | Material for glucose measurements | 0.24 | 1 | 23.52 |  |
|  | Lab technician’s working hours | 17.74 | 0.01 | 17.39 |  |

**Supplemental Data Figure S1.** Patient workflow

**
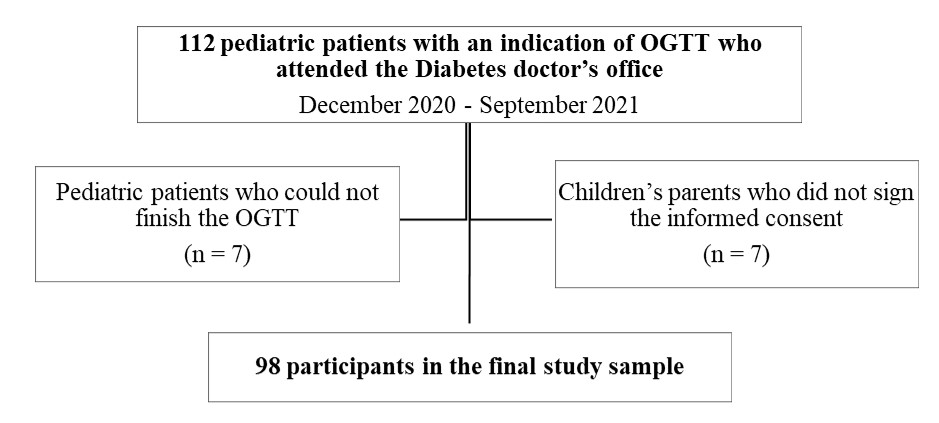
**

Abbreviations: OGTT, oral glucose tolerance test

**
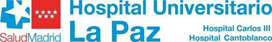
Supplemental Data File S1.** Satisfaction questionnaire for the Pediatric Endocrinology Department.

**SATISFACTION QUESTIONNAIRE FOR THE PEDIATRIC ENDOCRINOLOGY DEPARTMENT**

A research project called “Impact of incorporating point-of-care testing connected glucometers in the Pediatrics Endocrinology Department” has been conducted between the Clinical Analysis Department and Pediatric Endocrinology Department. The objective of the study was to evaluate the impact that incorporating these glucometers has had in terms of the correlation between results, turnaround time, diagnostic concordance, costs and staff satisfaction. We would therefore like to know how you would value the opportunity of using a connected glucometer (since December 2019), compared with the previous unconnected glucometer (before December 2019).

|  | Unconnected glucometer  (Before December 2019) | Connected  glucometer  (Since December 2019) | Score  (From 1 to 5) |
| --- | --- | --- | --- |
| Personal identification of trained and qualified clinical staff for using the POCT glucometer | X | ✓ | 4.4 |
| Patient identification with medical record number by a barcode | X | ✓ | 4.5 |
| Glucose strip lot identification by a barcode | X | ✓ | 4.6 |
| Possibility of choosing a predefined comment, specifying the functional test and time of the test | X | ✓ | 5 |
| Automatic transfer of patient results into the laboratory information system to generate a laboratory report in the electronic medical record | X | ✓ | 5 |
| Quality control material identification by a barcode | X | ✓ | 4.7 |
| Continuous monitoring by the laboratory medicine | X | ✓ | 5 |

The satisfaction questionnaire consists of three questions. Thank you for your collaboration.

1. What is your job?


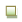

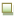


Nurse Doctor

2. In the table above, there are summarized the potential benefits of implementing a connected glucometer. Using values from 1 to 5, with 1 the most negative score and 5 the most positive. How would rate the benefit for each situation? If it does not apply, please indicate NA.

3. Would you recommend implementing connected glucometers in the same clinical context in other hospitals?

| 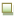 Yes | 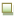No | 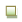 It depends |
| --- | --- | --- |

**Supplemental Data File S2.** Methods comparison between POCT_ACI_ and the central laboratory

**
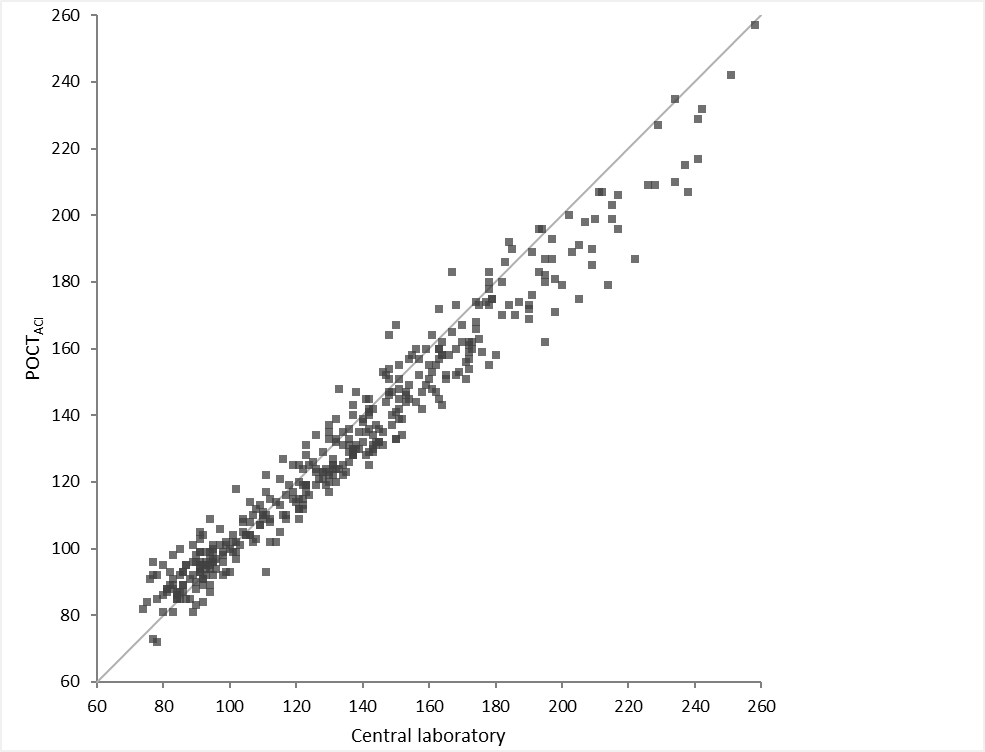
**
